# Perceived stress during the prenatal period: assessing measurement invariance of the Perceived Stress Scale (PSS-10) across cultures and birth parity

**DOI:** 10.1007/s00737-022-01229-5

**Published:** 2022-04-14

**Authors:** Laura Katus, Sarah Foley, Aja L. Murray, Bao-Yen Luong-Thanh, Diana Taut, Adriana Baban, Bernadette Madrid, Asvini D. Fernando, Siham Sikander, Catherine L. Ward, Joseph Osafo, Marguerite Marlow, Stefani Du Toit, Susan Walker, Thang Van Vo, Pasco Fearon, Sara Valdebenito, Manuel P. Eisner, Claire Hughes

**Affiliations:** 1 Centre for Family Research, University of Cambridge, Free School Lane, Cambridge, CB2 3RQ UK; 2 Department of Psychology, University of Cambridge, Cambridge, UK; 3 Moray House School of Education and Sport, University of Edinburgh, Edinburgh, UK; 4 Department of Psychology, University of Edinburgh, Edinburgh, UK; 5 Faculty of Public Health, Institute for Community Health Research, Hue University of Medicine and Pharmacy, Hue University, Hue, Vietnam; 6 Department of Psychology, Babes-Bolyai University, Cluj-Napoca, Romania; 7 Child Protection Unit, University of the Philippines, Manila, Philippines; 8 Colombo, Sri Lanka; 9 Global Health Department, Health Services Academy, Islamabad, Pakistan; 10 Department of Primary Care and Mental Health, University of Liverpool, Liverpool, UK; 11 Department of Psychology, University of Cape Town, Cape Town, South Africa; 12 Department of Psychology, University of Ghana, Accra, Ghana; 13 Department of Global Health, Institute of Life Course Health Research, Stellenbosch University, Cape Town, South Africa; 14 Caribbean Institute for Health Research, The University of the West Indies, Kingston, Jamaica; 15 Institute of Criminology, University of Cambridge, Cambridge, UK; 16 Jacobs Center for Productive Youth Development, University of Zurich, Zurich, Switzerland

**Keywords:** Pregnancy, Stress, Measurement invariance, Lower-middle income, Parity

## Abstract

Maternal prenatal stress places a substantial burden on mother’s mental health. Expectant mothers in low- and middle-income countries (LMICs) have thus far received less attention than mothers in high-income settings. This is particularly problematic, as a range of triggers, such as exposure to traumatic events (e.g. natural disasters, previous pregnancy losses) and adverse life circumstances (e.g. poverty, community violence), put mothers at increased risk of experiencing prenatal stress. The ten-item Perceived Stress Scale (PSS-10) is a widely recognised index of subjective experience of stress that is increasingly used in LMICs. However, evidence for its measurement equivalence across settings is lacking. This study aims to assess measurement invariance of the PSS-10 across eight LMICs and across birth parity. This research was carried out as part of the Evidence for Better Lives Study (EBLS, vrc.crim.cam.ac.uk/vrcresearch/EBLS). The PSS-10 was administered to *N* = 1,208 expectant mothers from Ghana, Jamaica, Pakistan, the Philippines, Romania, South Africa, Sri Lanka and Vietnam during the third trimester of pregnancy. Confirmatory factor analysis suggested a good model fit of a two-factor model across all sites, with items on experiences of stress loading onto a negative factor and items on perceived coping onto a positive factor. Configural and metric, but not full or partial scalar invariance, were established across all sites. Configural, metric and full scalar invariance could be established across birth parity. On average, first-time mothers reported less stress than mothers who already had children. Our findings indicate that the PSS-10 holds utility in assessing stress across a broad range of culturally diverse settings; however, caution should be taken when comparing mean stress levels across sites.

The United Nations Sustainable Development Goals identify the improvement of mental health as a key priority for global health and well-being (Izutsu et al. 2015). Globally, new mothers are amongst the most affected by poor mental health, with estimated depression rates ranging from 15.6 to 19.8% in the perinatal period (Atif et al. 2015). However, the field of maternal mental health is, like most psychological research, limited by a heavy focus on Western, Educated, Industrialised, Rich and Democratic countries (WEIRD, Henrich et al. 2010; Atif et al. 2015). As a result, commonly used assessment tools have been developed and normed almost exclusively in high-income settings. This is problematic as exposure to certain environmental stressors and poverty-related insecurities means that experiences of stress are as, if not more, severe in low- and middle-income countries (LMICs). Furthermore, commonly used mental health assessment tools may not carry the same meaning in cultures which were not involved in scale development and validation, meaning the scale may perform poorly in these settings.

A vital first step for extending the use of assessments of stress to LMICs is to scrutinise their measurement equivalence across settings. Increasingly, such investigations have been carried out in context of maternal and women’s mental health: for example, depression measures, such as Patient Health Questionnaire–9 (PHQ-9), have been examined in terms of its factor structure across expectant mothers in Peru (Smith et al. 2020), Spain (Marcos-Nájera et al. 2018) and China (Zheng et al. 2020),and the Self-Report Questionnaire (SRQ-20) is invariant in mothers across several LMICs (Pendergast et al. 2014). However, even in context of depression and even more so in context of perinatal stress, few studies to date provide much-needed rigorous, multi-site evaluations of commonly used assessment tools.

With this in mind, we examine the factor structure and measurement invariance (MI) of the ten-item version of the Perceived Stress Scale (PSS-10, Cohen et al. 1994) across eight LMICs. The original 14-item scale and its abbreviated ten- and four-item version have been used in a range of contexts (including pregnancy, Chaaya et al. 2010; Tanpradit and Kaewkiattikun 2020) and have undergone evaluation of their psychometric properties.

The overwhelming majority of studies assessing the factor structure of the PSS report best fit with a two-factor structure, specifically one latent factor on perceived stress and one on perceived coping (Taylor 2015; Lavoie and Douglas 2012; Reis et al. 2019). Conceptually, the PSS comprises items relating to both negative experiences of stress (e.g. ‘*…felt that you were unable to control the important things in your life ‘?*) and positive experiences of being able to cope with difficulties (e.g. ‘*…felt confident about your ability to handle your personal problems’?*). Though some have argued that the two factors are not truly independent but reflect differences in responses due to reverse coded items (e.g. Perera et al. 2017), there is an emerging consensus that items associated with perceived stress and reverse coded items measuring perceived coping are underpinned by separate latent factors (Taylor 2015). The PSS has been translated into over 25 languages (Lee 2012), demonstrating robust psychometric properties (e.g. good internal consistency, Cronbach’s alpha > 0.7, high longitudinal test–retest reliability intraclass correlation coefficient > 0.7). A two-factor structure was reported for versions in Greek (Andreou et al. 2011), Portuguese (Siqueira Reis et al. 2010), German (Bastianon et al. 2020), Chinese (Liu et al. 2020), Spanish (Juárez-García et al. 2021), Thai (Tanpradit and Kaewkiattikun 2020) and Arabic (Ali et al. 2021,). Moreover, MI has been demonstrated across men and women (Liu et al. 2020), longitudinally (Barbosa-Leiker et al. 2013; Reis et al. 2019), age groups and marital status (Ali et al. 2021). Nevertheless, questions regarding the structural and conceptual equivalence of the PSS in non-WEIRD settings remain. Firstly, a rigorous examination of the scale’s MI across multiple LMICs has not been conducted. Furthermore, despite their known vulnerability to mental health problems, expectant mothers situated in LMICs are currently understudied (Staneva et al. 2015), and questions remain whether stress levels differ between first-time mothers making the transition to parenthood compared to mothers of growing families. Addressing these twin gaps, the current study tests the factor structure and MI of the PSS-10 in expectant mothers across eight geographically and culturally diverse settings.

This research forms part of the Evidence for Better Lives Study (EBLS, vrc.crim.cam.ac.uk/vrcresearch/EBLS), which in its first wave has collected data from expectant mothers in Ghana, Jamaica, Pakistan, the Philippines, Romania, South Africa, Sri Lanka and Vietnam. In this analysis we aim to (1) perform confirmatory factor analysis (CFA) to assess whether a previously reported two-factor model provides a good fit and (2) test the assumptions of MI (i.e. configural, metric, scalar) across site and birth parity, to assess whether the same underlying constructs are tapped across study settings and across first- and none-first-time mothers. Such an analysis provides a crucial step when seeking to investigate the cultural universality of the measure and a prerequisite for meaningful cross-site comparisons.

## Methods

### Participants

Assessments took place as part of the EBLS project, a prospective longitudinal cohort study examining *N* = 1,208 families across eight LMICs. Ethics boards at each participating institution approved the protocol (Valdebenito et al. 2020).

Expectant mothers were recruited during routine antenatal clinic visits from primary healthcare facilities. They were eligible if they (1) were in the third trimester of pregnancy (29–40 weeks gestation), (2) aged 18 or over and (3) living primarily within the study’s catchment area. On average, 82% of women approached consented to participate. Sample characteristics can be found in Table 1.

**Table 1:** Descriptive statistics (maternal age and years in education and proportion of first-time mothers per site)

	Koforidua, Ghana *n* = 150	Kingston, Jamaica *n* = 152	Tarlai Kalan, Pakistan *n* = 150	Valenzuela, Philippines *n* = 154	Cluj-Napoca, Romania *n* = 150	Worcester, South Africa *n* = 150	Ragama, Sri Lanka *n* = 152	Hue, Vietnam *n* = 150	Between-site difference	
Language of administration	Twi	English	Urdu	Tagalog	Romanian	Afrikaans and IsiXhosa	Sinhala and Tamil	Vietnamese		
	*x̄* ± *SD*	*x̄* ± *SD*	*x̄* ± *SD*	*x̄* ± *SD*	*x̄* ± *SD*	*x̄* ± *SD*	*x̄* ± *SD*	*x̄* ± *SD*	*F*	*η*^*2*^
Maternal age (years)	29.07 ± 6.314	25.53 ± 5.687	27.29 ± 5.187	27.66 ± 6.039	30.01 ± 4.617	26.95 ± 5.955	29.74 ± 5.534	29.94 ± 5.146	13.29***	0.072
Education level (years completed)	8.38 ± 4.327	10.46 ± 1.054	7.77 ± 4.791	11.42 ± 2.649	12.83 ± 1.971	10.48 ± 1.892	11.85 ± 1.464	10.54 ± 2.477	51.379***	0.240
									*χ*^*2*^	*Cramer’s V*
Proportion of nulliparous participants	15.7%	39.6%	27.7%	16.8%	64.9%	38.1%	42.5%	56.6%	92.571***	1.2079

*** *p* < 0.001

Participants’ average age was 28.27 years (range = 18–48 years): women in Ghana, the Philippines, Romania, Sri Lanka and Vietnam on average were older than women in Jamaica, South Africa and Pakistan. Thirty percent of women were nulliparous, with higher rates in Romania (64.9%) and lowest rates in Ghana (15.7%). Education levels ranged from 0 to 20 years completed, with an average of 7.77 years in Pakistan (Anwer et al. 2022) and 12.83 years completed in Romania. For this analysis, data of women expecting twins were retained.

### Procedure

Interviews took place from December 2018 to July 2019. Participants provided written or audio-recorded informed consent. During their third trimester of pregnancy, expectant mothers were interviewed by trained fieldworkers using primarily computer-assisted personal interviews (CAPI). Training sessions, to ensure consistency across the study sites and adherence to ethical, health and safety requirements, included the coordination and management tasks, recruitment, sampling, ethics and questionnaire administration. All training resources were combined into a fieldworker manual as a reference during data collection. Interviews were generally conducted alongside routine antenatal care appointments, in a separate room to ensure privacy.

### Measures

#### PSS-10

Participants completed the PSS-10 antenatally as part of an extensive questionnaire battery. The full protocol included questionnaires on participants’ physical and mental health, exposure to adversity, social support, attitudes about their pregnancy and parenting and reproductive history. Participants responded to negative (e.g. ‘…been upset because of something that happened unexpectedly’?) and positive items (e.g. ‘…felt confident about your ability to handle personal problems’?) pertaining to the levels of stress and coping over the past month. Responses were given on a four-point Likert scale (1 = not at all, 2 = several days, 3 = more than half the days, 4 = nearly every day). This differed from the usual PSS-10 response scale (five-point Likert scale, 1 = never–5 = very often), to harmonise response options of the PSS-10 with those of the PHQ-9. This harmonisation was necessary, as the full protocol included 19 measures with a total of 212 items, meaning that a retention of each scale’s original response format would have required frequent response-mode-switches. Given varying levels of familiarity with standardised questionnaires, participant literacy and the resulting need to administer the questionnaires verbally, response anchors were harmonised wherever possible.

#### Secondary outcomes

Participants reported demographic information, including their age, socioeconomic status, highest level of education, number of previous pregnancies and live births.

### Translation and piloting

Study materials were translated into the most common languages spoken by participants, guided by the Translation Review Adjudication Pretest Documentation (TRAPD) method (https://europeanvaluesstudy.eu/methodology-data-documentation/survey-2017/methodology/the-trapd-method-for-survey-translation/). Translations followed the same process across study sites to ensure maximal consistency. Where measures had previously been translated into the relevant languages, we conducted our own translations to ensure consistency. In Jamaica, the original English language version was used with slight adaptations. Harmonised translation was achieved through two independent forward translations, which were reviewed by expert panels at each study site. These panels comprised staff who were knowledgeable regarding both the measures employed and cultural views on mental health. Measures were piloted on *n* = 5–10 women per site to identify and correct issues with comprehension or translation. Pilots revealed minor ambiguities within the full protocol, but not the PSS-10. Prior to the start of data collection, field workers were trained to address potential ambiguities during administration.

### Data analysis

#### Data screening

Data were analysed in Mplus 8.4 (Muthén and Muthén 1998–2017). Responses were clustered around the upper and lower end of the response scale, necessitating the dichotomisation of the response options (i.e. 0 = *not at all/several days*, 1 = *more than half the days/nearly every day*, Rutkowski et al. 2019). This approach led to a floor effect on one item in the Romanian cohort (1, ‘*…been upset because of something that happened unexpectedly’?*), which was therefore removed. Therefore, analyses included five negative and four positive items. Analyses applied weighted least squares mean and variance adjusted (WLSMV) estimators as items were ordered categorical.

#### Measurement invariance across sites

We first assessed the factor structure of the PSS-10 per site. Our model fit criteria were Comparative Fit Index (CFI) > 0.90, Tucker Lewis Index (TLI) > 0.90 and Root Mean Square Error of Approximation (RMSEA) < 0.08 (Brown 2015; Hu and Bentler 1999). We then examined the change in model fit when systematically adding equality constraints. Model comparisons were judged to be invariant if the CFI decreased < 0.02 and the RMSEA increased < 0.003 (Svetina et al. 2020). Since no group was chosen as a reference in designing the study, we used the site appearing first in the alphabet (i.e. Ghana) as our reference group, in which the mean of the latent factor was fixed to 0 and the variance of the latent factor and scale factor were fixed to 1. Where model fit was low, we examined modification indices to identify reasons for poor fit. Assuming a reasonable fit for each group, we proceeded to test the configural invariance across sites (i.e. whether a common factor structure could be found across sites). Next, we examined metric (weak factorial) invariance, by constraining the factor loadings to be equal across groups (i.e. we assessed whether items contributed to each factor in a similar way across sites). Where metric invariance could be established, we proceeded to test scalar (strong factorial) invariance, to compare whether item thresholds were equivalent. If full scalar invariance was not achieved, constraints on the model were released on an item-by-item basis across all groups to identify a partial scalar invariant subset of items. Where full or partial scalar invariance could be achieved, we compared mean levels for each latent factor across sites.

#### Measurement invariance across birth parity

We split the sample, grouping together women expecting their first (nulliparous group) vs those expecting a subsequent child (multiparous group). As described above, we then conducted CFA and tested configural, metric and scalar invariance across groups.

## Results

### PSS factor structure by site

The hypothesised two-factor solution presented an acceptable fit for all sites (Table 2). A one-factor model resulted in poor model fit and was therefore not taken forward.

**Table 2:** Model fit indices for 2-factor model of the Perceived Stress Scale by study site

Site	RMSEA		CFI	TLI
	Estimate	95% confidence interval		
Ghana	0.091	0.059-0.123	0.920	0.889
Jamaica	0.000	0-0.063	1	1
Pakistan	0.027	0-0.073	0.985	0.98
Philippines	0.080	0.047-0.112	0.961	0.946
Romania	0.069	0.032-0.103	0.964	0.950
South Africa	0.059	0.011-0.094	0.945	0.924
Sri Lanka	0.000	0-0.064	1	1
Vietnam	0.000	0-0.057	1	1

*RMSEA* Root Mean Square Error of Approximation, *CFI* Comparative Fit Index, *TLI* Tucker Lewis Index

### Measurement invariance in prenatal stress across sites

We first assessed the configural invariance by site, yielding a good model fit (RMSEA = 0.058, CI_95%_ = 0.035–0.064; CFI = 0.963; TLI = 0.962). Constraints to test for metric invariance did not significantly reduce the model fit (RMSEA = 0.06, CI_95%_ = 0.048–0.071; CFI = 0.949; TLI = 0.946), however constraints to test for scalar invariance did (RMSEA = 0.127, CI_95%_ = 0.118–0.136; CFI = 0.774; TLI = 0.779). Modification indices suggested to release constraints for six items. However, the three remaining items (2 ‘…were unable to control the important things in your life’?, 3 ‘…felt nervous and ‘’stressed’’’?, 10 ‘…felt difficulties were piling up so high that you could not overcome them’?) still did not produce an acceptable model fit (RMSEA = 0.088, CFI = 0.886; TLI = 0.875). This lack of scalar invariance indicated that mean differences in the latent variable did not capture all shared variance across items, precluding a comparison of mean levels across sites.

### Measurement invariance and mean differences in prenatal stress across birth parity

Testing the two-factor model’s configural invariance across nulliparous and multiparous women revealed a good model fit (RMSEA = 0.043, CI_95%_ = 0.031–0.054; CFI = 0.971; TLI = 0.960). Adding metric (RMSEA = 0.044, CI_95%_ = 0.033–0.054; CFI = 0.966; TLI = 0.959) and scalar (RMSEA = 0.044, CI_95%_ = 0.034–0.054; CFI = 0.963; TLI = 0.958) constraints resulted in an equally good model fit. We therefore compared means of the latent positive and negative factors across groups. Both factors showed significantly higher mean levels in the multiparous compared to the nulliparous group (b_negative_ = 0.064, SE = 0.014, *p* < 0.001, b_positive_ = 0.073, SE = 0.023, *p* = 0.002).

## Discussion

The current study assessed MI of the PSS-10 in *N* = 1,208 expectant women across eight LMICs. While the detrimental effects of poor mental health for mothers and children are well-documented (e.g. Karam et al. 2016), the literature is skewed towards high-income settings. We found configural and metric MI across sites and configural, metric and scalar MI across birth parity. PSS mean levels were higher for both the positive and the negative factor in mothers who already had at least one child.

### Factor structure and response mode

In line with previous studies (Reis et al. 2019; Bastianon et al. 2020; Liu et al. 2020; Juárez-García et al. 2021; Ali et al. 2021), we found strong evidence for a two-factor (one positive, one negative) structure across sites and parity. Responses were clustered towards the extremes of the scale, necessitating dichotomisation of items. As such a bimodal distribution is uncommon for the PSS, future research should investigate possible reasons in context of diverse non-WEIRD settings. Practically, this finding suggests a dichotomised response format may be favourable in LMICs.

### Cross-site comparison

We found metric invariance across sites, with items loading onto the latent factors in a similar manner. However, the lack of scalar invariance precluded meaningful cross-site comparisons. The inability to reach this threshold was especially clear for the positive factor (c.f., Santiago et al. 2020). Additionally, some have argued that positive and negative mental health factors represent distinct concepts, meaning that differences in their psychometric properties may be attributable to them capturing different constructs (Phua et al. 2020). Therefore, reporting and analysing scores on both subscales separately may be favourable. We also explored the possibility that differences in response patterns across sites may reflect idiosyncrasies in some positive items (e.g. 5 ‘*…felt that things were going your way’?, 8 ‘…felt that you were on top of things’?*). However, a review of these items by site-specific experts indicated that these items had not been identified as problematic. Recent investigations from within our group (e.g., on the prenatal attachment index, Foley et al. 2021 and the PHQ-9, Murray et al. 2021), and in the wider literature (Dong and Dumas 2020) also find that the more stringent criteria of MI cannot always be met in cross-cultural research. In the context of perinatal mental health, this perhaps can be attributed to the fact that different ways of coping lead to similar outcomes across cultures (Guardino and Dunkel Schetter 2014). For example, mothers’ acceptance of one’s situation was associated with positive pregnancy outcomes in the USA, and Japanese mothers benefitted more from greater social assurance (Morling et al. 2003). Thus, even where the item structure and factor loadings are equivalent, different endorsement of specific items (e.g. on culturally specific coping styles) may lead to a lack of equivalence in item thresholds.

### Comparison across birth parity

We found full scalar MI of the PSS-10 across these two groups. Multiparous mothers showed higher mean levels on both latent factors. One reason for this may be an increased awareness of pregnancy stressors and their own coping ability. In high-income settings, while the transition to parenthood in first-time parents is regarded as the more dramatic change, the addition of subsequent children is associated with higher levels of stress (Gameiro et al. 2009). Further corroborating the link between parity and maternal stress, prenatal exposure to environmental adversity has been shown to affect first-time mothers most severely (Terán et al. 2020). Less attention has been paid to perceived control and self-efficacy; however, these may be higher in multiparous mothers (Loh et al. 2017).

### Limitations and strengths

Responses necessitated the use of a dichotomised scale which can inflate estimates of model fit (Rutkowski et al. 2019). This highlights certain drawbacks of Likert scales across different cultures, as cultural factors can contribute to bimodal distributions (Lee et al. 2002). Our other analyses (Foley et al. 2021; Murray et al. 2021) have suggested that a dichotomised administration may be favourable. This is especially true given that questionnaire-based mental health measures developed in high-income settings often show poorer reliability in LMICs (Carroll et al. 2020; Shrestha et al. 2016) and would benefit from site-specific validation against both subjective self-report and objective biological measures (e.g. measures of cortisol).

Harmonising response formats and simplifying responses for participants, we adopted the response scale of the PHQ-9, which uses similar anchors and captures the intensity and frequency. Considering the post hoc dichotomisation in the current and recent studies by our group (Foley et al. 2021; Murray et al. 2021), and in the wider literature (e.g. General Health Questionnaire (GHQ), Goldberg 1988) and the practicalities of administration, future studies may consider dichotomising response options, especially in context of multi-questionnaire, multi-site research. While administrators were trained with the greatest care to ensure reliability, a simplified dichotomised format may serve to further eliminate site differences.

Non-random sampling was applied, limiting the sample’s representativeness. However, across sites several diverse contexts were covered. Our sample sites differed regarding parity and maternal age. Here, differences in family structure need to be acknowledged: while of similar mean age, only 15.7% of women in Ghana were expecting their first child, compared with 64.9% of mothers in Romania. It therefore was not possible to achieve homogeneity across both age and parity, and a lack of MI across sites may in part be attributable to these differences. Furthermore, the potential for cultural differences regarding the understanding of the PSS and the expression of experiences of stress have been highlighted in more qualitative ways in other cross-cultural studies (Ting et al. 2021), and such differences may also have contributed to our results. Lastly, it needs to be noted that formal evaluation of other forms of validity and reliability of the PSS was beyond the scope of this study.

## Conclusion and Future Directions

While we have established that the PSS-10 follows a reliable structure across settings, questions remain regarding the optimal response format for use in LMICs. A dichotomised scoring approach may be favourable, given issues with floor and ceiling effects. Our differential findings on the reliability of the positive and the negative subscale may warrant independent reporting of scores on each subscale, to examine possible differential associations of perceived stress and perceived coping with participant outcomes. The PSS-10 showed good configural and metric fit cross-culturally, highlighting its utility for use across a broad range of settings. Caution is advised when comparing mean levels of perceived stress across settings. Regarding birth parity, the PSS-10 passes all tests of conceptual equivalence, enabling mean level comparisons across birth parity.
